# Useful resources for cataract complications

**Published:** 2008-03

## Community Eye Health Journal back issues

**Sandford-Smith J. Sutureless cataract surgery: principles and steps.** Comm Eye Health J 2003;16(48): 51–53.

**Schroeder B. Sutureless cataract extraction: complications, management and learning curves.** Comm Eye Health J 2003;16(48): 58–59.

## Books

**Noble BA and Simmons IG. Complications of cataract surgery: a manual.** 2nd ed. Butterworth Heinemann, 2001. Available from Waterstones. UK £54.99 plus post and packing.

**Sandford-Smith J. Eye surgery in hot climates.** 2nd ed. UK £9 (plus postage and packing). Available from the International Centre for Eye Health.

## Other resources

**Hennig A and Schroeder B. Sutureless cataract surgery: ‘fishhook technique’ instruction course.** CD-ROM. Available free of charge from CBM.

**Sandford-Smith J. Extracapsular cataract extraction with IOL implantation for developing countries.** Video. UK £5 (incl. post and packing). Available from the International Centre for Eye Health.

**Stevens S. Control of infection in ophthalmic practice.** Poster. **Cox I and Stevens S. Sterilisation and disinfection.** Poster.

Both available from the International Centre of Eye Health. Free of charge to low- and middle-income countries, UK £3 otherwise.

## Suppliers' addresses

**CBM:** Email procurement@cbm.org or write to CBM Procurement, Christian Blind Mission e.V., Nibelungenstrasse 124, 64625 Bensheim, Germany.

**Waterstones:** 71–74 North Street, Brighton, East Sussex BN1 1ZA, UK. Email: manager@brighton.waterstones.co.uk

**International Centre for Eye Health:** Write to Jenni Sandford, London School of Hygiene and Tropical Medicine, Keppel Street, London WC1E 7HT, UK. Email: jenni.sandford@Lshtm.ac.uk

